# Neurotrophins are expressed in giant cell arteritis lesions and may contribute to vascular remodeling

**DOI:** 10.1186/s13075-014-0487-z

**Published:** 2014-11-24

**Authors:** Kim Heang Ly, Alexis Régent, Elsa Molina, Sofiane Saada, Philippe Sindou, Claire Le-Jeunne, Antoine Brézin, Véronique Witko-Sarsat, François Labrousse, Pierre-Yves Robert, Philippe Bertin, Jean-Louis Bourges, Anne-Laure Fauchais, Elisabeth Vidal, Luc Mouthon, Marie-Odile Jauberteau

**Affiliations:** Laboratoire d’immunologie, EA3842, Faculté de Médecine, Université de Limoges, 2 rue Dr Marcland, Limoges, 87025 France; Service de Médecine Interne A, CHU Dupuytren, 2 avenue Martin Luther King, Limoges, 87042 France; Institut Cochin, INSERM U1016, CNRS UMR 8104, Université Paris Descartes, 22 rue Méchain, Paris, 75014 France; Service de Médecine Interne, Centre de Référence pour les vascularites nécrosantes et la sclérodermie systémique, Hôpital Cochin, Assistance Publique Hôpitaux de Paris (AP-HP), 27 rue du Faubourg Saint-Jacques, Paris, 75014 France; Service de Médecine Interne, Hôpital Hôtel Dieu, AP-HP, 1, place du Parvis de Notre Dame, Paris, 75181 France; Centre d’ophtalmologie, HUPC Cochin Hôtel-Dieu, Université Sorbonne Paris Cité, Faculté de Médecine Paris Descartes, AP-HP, 27 rue du Faubourg Saint-Jacques, Paris, 75014 France; Service d’Anatomie Pathologique, CHU Dupuytren, 2 avenue Martin Luther King, Limoges, 87042 France; Service d’Ophtalmologie, CHU Dupuytren, 2 avenue Martin Luther King, Limoges, 87042 France; Service de Rhumatologie, CHU Dupuytren, 2 avenue Martin Luther King, Limoges, 87042 France

## Abstract

**Introduction:**

Giant cell arteritis (GCA) is characterized by intimal hyperplasia leading to ischaemic manifestations that involve large vessels. Neurotrophins (NTs) and their receptors (NTRs) are protein factors for growth, differentiation and survival of neurons. They are also involved in the migration of vascular smooth muscle cells (VSMCs). Our aim was to investigate whether NTs and NTRs are involved in vascular remodelling of GCA.

**Methods:**

We included consecutive patients who underwent a temporal artery biopsy for suspected GCA. We developed an enzymatic digestion method to obtain VSMCs from smooth muscle cells in GCA patients and controls. Neurotrophin protein and gene expression and functional assays were studied from these VSMCs. Neurotrophin expression was also analysed by immunohistochemistry in GCA patients and controls.

**Results:**

Whereas temporal arteries of both GCA patients (*n* = 22) and controls (*n* = 21) expressed nerve growth factor (NGF), brain-derived neurotrophic factor (BDNF), tropomyosin receptor kinase B (TrkB) and sortilin, immunostaining was more intense in GCA patients, especially in the media and intima, while neurotrophin-3 (NT-3) and P75 receptor (P75^NTR^) were only detected in TA from GCA patients. Expression of TrkB, a BDNF receptor, was higher in GCA patients with ischaemic complications. Serum NGF was significantly higher in GCA patients (*n* = 28) vs. controls (*n* = 48), whereas no significant difference was found for BDNF and NT-3. NGF and BDNF enhanced GCA-derived temporal artery VSMC proliferation and BDNF facilitated migration of temporal artery VSMCs in patients with GCA compared to controls.

**Conclusions:**

Our results suggest that NTs and NTRs are involved in vascular remodelling of GCA. In GCA-derived temporal artery VSMC, NGF promoted proliferation and BDNF enhanced migration by binding to TrkB and p75^NTR^ receptors. Further experiments are needed on a larger number of VSMC samples to confirm these results.

**Electronic supplementary material:**

The online version of this article (doi:10.1186/s13075-014-0487-z) contains supplementary material, which is available to authorized users.

## Introduction

Giant cell arteritis (GCA) is a primary vasculitis involving aorta and its extra- and intracranial branches. Histopathological findings show cellular infiltrates, internal elastic lamina disruption and intimal hyperplasia, leading to luminal stenosis. Macrophages and giant cells produce platelet-derived growth factor (PDGF), which stimulates migration of vascular smooth muscle cells (VSMCs) from the media to the intima to initiate intimal hyperplasia. However, several mechanisms involved in vascular remodelling of GCA still remain unclear [1].

Neurotrophins (NTs) are growth factors initially described in the nervous system and now include vascular cells [2]. Three NTs and their receptors (NTRs) are well investigated. They include the nerve growth factor (NGF), brain-derived neurotrophic factor (BDNF) and neurotrophin-3 (NT-3), the selective tropomyosin receptor kinase (Trk), TrkA for NGF, TrkB for BDNF and TrkC for NT-3, a non-selective receptor p75^NTR^ and a co-receptor, which is usually associated with p75^NTR^, called sortilin. In the cardiovascular system, NTs and NTRs are involved in cardiovascular development, blood vessel growth, VSMCs and cardiomyocyte functions [2]. NTs and NTRs are detected *in vivo* and *in vitro* in endothelial cells and human aortic VSMC donors and in rats [3]. In addition, NGF promotes similar VSMC migration as PDGFs, but not their proliferation [3,4]. Moreover, NGF and BDNF and their two specific Trk receptors are involved in aortic intimal hyperplasia induced by balloon angioplasty in rats [3], while activation of p75^NTR^ by NGF, NT-3 and, to a lesser extent, BDNF, induce VSMC apoptosis [5].

Using temporal artery VSMCs (TASMCs) obtained prospectively from suspected GCA patients, we hypothesized that NTs and NTRs are involved in the pathogenesis of GCA, especially in the vascular remodelling stage.

## Methods

### Patients

We prospectively enrolled patients who had undergone a temporal artery biopsy (TAB) for suspected GCA at Limoges and Paris (Cochin and Hotel-Dieu) university hospitals, since January 2010. GCA diagnosis was established according to American College of Rheumatology (ACR) criteria and biopsy-proven GCA lesions [6]. Patients with a negative TAB without clinical diagnosis of arteritis were defined as controls. All subjects gave their informed consent prior to participation and the study was approved by the Ethics Committee of the Cochin University of Paris (collection dc-2010-1079). Clinical and biological data are shown in Table 1.

**Table 1:** **Clinical and biological findings in the study cohort patients with GCA**

**Clinical characteristics**	**GCA patients (Plasma study) N (%)**	**GCA patients (IHC study) N (%)**	**GCA patients (Cohort study) N (%)**
Number of patients	30	22	42
**General characteristics**			
Age in years, median (range)	77 (60-92)	80.7 (73-89)	76.3 (60-92)
Sex, women/men	23/7	14/8	30/12
**Systemic manifestations**	15 (50)	17 (77)	33 (78.5)
Fever	9 (30)	4 (18)	13 (31)
Weight loss	15 (50)	12 (54.5)	20 (47.6)
**Cranial symptoms**	21 (70)	14 (63.6)	31 (73.8)
Headache	21 (70)	14 (63.6)	31 (73.8)
Jaw claudication	15 (50)	11 (50)	19 (45.2)
Scalp tenderness	14 (46.6)	12 (54.5)	21 (50)
Abnormal temporal arteries^*^	13 (43)	7 (32)	7 (16.6)
**Cranial ischaemic events**	9 (30)	8 (36)	11(26)
Permanent visual loss	1 (3)	3 (13.6)	2 (4.7)
Amaurosis fugax	6 (20)	8 (36)	11 (26)
Stroke	2 (6.6)	1 (4.5)	3 (7)
**Polymyalgia rheumatica**	7 (23)	7 (32)	10 (24)
**Biological findings**	**Mean ± SD**	**Mean ± SD**	**Mean ± SD**
Erythrocyte sedimentation rate (mm/h)	85.8+/-25	84+/-28	77.8+/-26
C-reactive protein (mg/L)	102.2+/-70.3	92.6+/-66	86.2+/-78
Platelet count (x 10^9^/L)	476+/-157	484+/-162	448+/-150
Haemoglobin (g/L)	11.5+/-1.6	12.2+/-1.4	11.8+/-1.7

^*^Abnormal temporal arteries at physical examination (painful, swollen, indurated and/or with decreased or absent pulsation). GCA, giant cell arteritis; IHC, immunohistochemistry; SD, standard deviation.

### Cell culture

TASMCs were isolated from segments of 5 to 10 mm of temporal artery (TA) after removal of adventitia and enzymatic digestion of media with 500 U/mL of type I collagenase in 5 mL of Dulbecco’s modified Eagle’s medium (DMEM) Glutamax II (Gibco, Grand Island, NY, USA). Cultures were performed, as previously described [7], in Smooth Muscle Cell Basal Medium (PromoCell, Heidelberg, Germany) supplemented with foetal calf serum (FCS) (5%), insulin (5 μg/mL), fibroblast growth factor (FGF-2) (2 ng/mL), epidermal growth factor (0.5 ng/ml) and streptomycin/penicillin (1%) at 37°C in 5% CO_2._ Functional assay cells were growth factor-starved and maintained in DMEM-free medium for 24 h before stimulation.

Cultures contained 90% VSMC cells expressing α-smooth-muscle actin (Sigma-Aldrich, St Louis, MO, USA), without CD-90 fibroblastic staining (Dianova, Hamburg, Germany) (Additional file 1). TASMCs were used for the experiments between passages 3 and 8. The cells were studied for NTs and NTRs expression at protein and transcriptional levels and in proliferation and migration assays. GCA-derived TASMCs used for western blotting, real-time polymerase chain reaction (PCR) analysis and functional assays were obtained from patients with positive TAB for GCA.

### Immunohistochemistry

Immunohistochemistry was performed on paraffin-embedded sections of TA deparaffinised in 22 biopsy-proven GCA patients according to previous described criteria [8] and 21 controls with rabbit anti-NGF, -BDNF, -NT-3, -TrkA, -TrkB, -TrkC, -p75^NTR^ and goat anti-sortilin antibodies (Ab) (Santa Cruz, Heidelberg, Germany) at 1:100 and anti-CD3 Ab for T lymphocytes (1/400; Dako Glostrup, Denmark). Sections of TA were rehydrated and subjected to steam-heat antigen retrieval in citrate buffer in a microwave oven (750 W). Endogenous peroxidases were inhibited with 5% H_2_O_2_ in methanol and non-specific sites were blocked with phosphate-buffered saline (PBS)-3% bovine serum albumin. The primary Ab as above were incubated at a 1/100 dilution overnight at 4°C and revealed with the anti-rabbit HRP Envision™ (Dako). After counterstaining, the slides were studied with a Leica microscope (×200 magnification) (Leica, Wetzlar, Germany). Immunostaining was scored using a staining index based on the percentage of positive cells. Positive controls were obtained from neuroblastoma and breast cancer tissues. Negative controls were done with isotypic immunoglobulin controls (data not shown). To perform semi-quantitative measurement of immunostaining, we chose three representative area of temporal artery (×200 magnification) to count staining cells that expressed myofibroblast or VSMC phenotype (that is, elongated cells). The mean of positive cell percentage of these three measurements for each patient or control was used to determine immunostaining intensity. The results were expressed as the mean of three independent quantifications made by two different observers.

### Western blotting

Western blotting was performed as previously described [9] with rabbit anti-NGF, -BDNF, -NT-3, -TrkA, -TrkB, -p75^NTR^ and goat anti-sortilin Ab (Santa Cruz) or anti-TrkC (R&D Systems, Minneapolis, MN, USA) at 1/200 dilution. Signalling activation was studied by using rabbit anti-protein kinase B (Akt) and -phospho-Akt Ab and -phospho-extracellular signal-regulated kinase (Erk)1/2 and mouse anti-Erk1/2 Ab (at 1/1000 dilution; R&D Systems). Proteins (50 μg/lane) obtained from whole-cell lysates of TASMCs using lysis buffer [9] were separated on 10 to 12% SDS-polyacrylamide gels (Invitrogen, Carlsbad, CA, USA) and transferred onto PVDF membranes (Merck Millipore, Billerica, MA, USA). After saturation (5% nonfat dry milk, 0.1% Tween in PBS), Ab were incubated overnight at 4°C, and revealed after 1 h incubation at room temperature (RT) with anti-rabbit or -goat Ig-HRP-conjugated Ab (Santa Cruz, dilution 1/1000) by chemiluminescence (ECL reagent; Amersham, Little Chalfont, UK). Protein-loading control was performed with anti-actin Ab (Santa Cruz, 1/10,000). Western blots were scanned using a bioimaging system (Genesnap; Syngene, Cambridge, UK).

### RNA extraction, reverse transcription and real-time PCR assays

Total RNAs were extracted with Qiagen RNeasy Isolation Kit (Qiagen, Venlo, Netherlands), treated with RNase-free DNase I (Qiagen), and quantified by NanoDrop (ND-1000) spectrophotometer (NanoDrop, Wilmington, DE, USA). The RNA quality was evaluated on an Agilent 2100 bioanalyzer using the RNA 6000 Labchip kit (Agilent Technologies, Palo Alto, CA, USA). cDNA synthesis was performed with a High-Capacity cDNA Reverse Transcription Kit (Applied Biosystems, Carlsbad, CA, USA), as recommended by the manufacturer. Real-time PCR was performed by predesigned primer/probe TaqMan gene expression assays (Applied Biosystems) and normalized to HPRT housekeeping gene (Additional file 2). Experiments were assessed in triplicate at least using TaqMan Fast-Univ Master Mix (Applied Biosystems) using an Applied Biosystems StepOnePlus Real-Time PCR System and analysed by StepOne Software v2.2.2 with a two-step PCR protocol as previously described [9]. The comparative ΔCt method was used for relative quantification of gene expression on duplicate of each reaction.

### Cell viability, proliferation and chemoinvasion assays

Cell viability, proliferation and migration assays were studied using exogenous NGF (Alomone Labs, Jerusalem, Israel), recombinant human BDNF (Promega, Madison, WI, USA), or PDGF-AB (R&D Systems, Minneapolis, Minnesota, USA), alone or both with K252a, a pharmacological Trk inhibitor (Alomone), ANA-12 (100 μM), a specific TrkB inhibitor, acting without altering TrkA and TrkC functions (Tocris, Bristol, UK) [10] or an anti-p75^NTR^ antagonist Ab (Alomone).

Cell viability of TASMCs was assessed using the colorimetric XTT assay (Cell Proliferation Kit II, XTT, Roche, Basel, Switzerland) according to the manufacturer’s instructions. A range of concentrations was evaluated for each of the conditions studied (Additional file 3). Efficient concentrations were defined as follows: NGF (10 ng/mL), BDNF (200 ng/mL), K252a Trk inhibitor (100 ng/mL), ANA-12 (100 μM), anti-p75^NTR^ antagonist Ab (10 mg/mL) and PDGF (40 ng/mL), which was used as a positive control [11].

Cell proliferation was determined by incorporation of BrdU (Cell Signaling, Danvers, MA, USA) according to the manufacturer’s instructions. Assays were studied using recombinant human NGF (10 ng/mL), recombinant human BDNF (200 ng/mL), or recombinant human PDGF-AB (40 ng/mL), alone or both with K252a Trk inhibitor (100 ng/mL), or ANA-12 specific TrkB inhibitor (100 μM) or an anti-p75^NTR^ antagonist Ab (10 mg/mL). Efficient concentrations of exogenous NTs, PDGF and NT receptor inhibitors were previously determined with XTT assay. Cells were seeded in 96-well plates (5,000 cells/well) and maintained in DMEM-free serum medium during 48 h before stimulation. Proliferation assays were performed after one, three and four days of TASMC incubation with NT or NTR inhibitors, or both.

### Chemoinvasion assay

The Boyden chamber method was performed in a BD Biocoat Matrigel Invasion Chambers (BD Biosciences, Franklin Lakes, NJ, USA) with an 8-μm pore-size membrane for the chemoinvasion assay. Experiments were performed in duplicate in at least two independent experiments. Serum-starved TASMCs were seeded (1 × 10^5^ cells/well) in 500 μL of serum-free DMEM into the upper chamber of the membrane-embedded insert during 48 h before stimulation. DMEM containing either 10% FCS alone or NGF, BDNF or PDGF at the concentrations defined above were placed in the lower chamber and the cells were incubated for 24 or 48 h at 37°C/5% CO_2_. After incubation, the lower chamber was treated with 50 μM Calcein AM fluorescent dye for 30 min at 37°C/5% CO_2_. Non-invading cells in the upper part of the insert were carefully removed. Then, 8 μl of Calcein AM fluorescent dye (BD Biosciences) was added for 15 min to the lower chamber. Non-invading cells in the upper part of the insert were carefully removed. The migration of TASMCs to the lower part was quantified by fluorescent counting spectrophotometry at 488 nm (Berthold, Bad Wildbad, Germany) and images were obtained using a fluorescence microscope M2FLIII (Leica).

### Measurement of sera and supernatant neurotrophin secretion

Cell-culture medium was collected and centrifuged for 30 min at 3,000 g in Vivaspin columns (Merck Millipore). Concentrations of NGF, BDNF and NT-3 in TASMC supernatant and 1:100 diluted sera from GCA patients and controls were measured using enzyme-linked immunosorbent assay (ELISA) kits according to the manufacturer’s instructions (NGF or BDNF or NT-3 EmaxH ELISA, Promega).

### Statistical analysis

Results were expressed as their means ± standard errors of the mean (SEM) of at least three independent experiments. Statistical analyses were performed using Student’s *t* test; a *P* value <0.05 was considered statistically significant. Asterisks in figures indicate statistically significant differences: ^*^P <0.05, ^**^P < 0.01, ^***^P <0.001.

## Results

### Neurotrophins are overexpressed in temporal arteries from patients with GCA compared to controls

**Figure 1:**
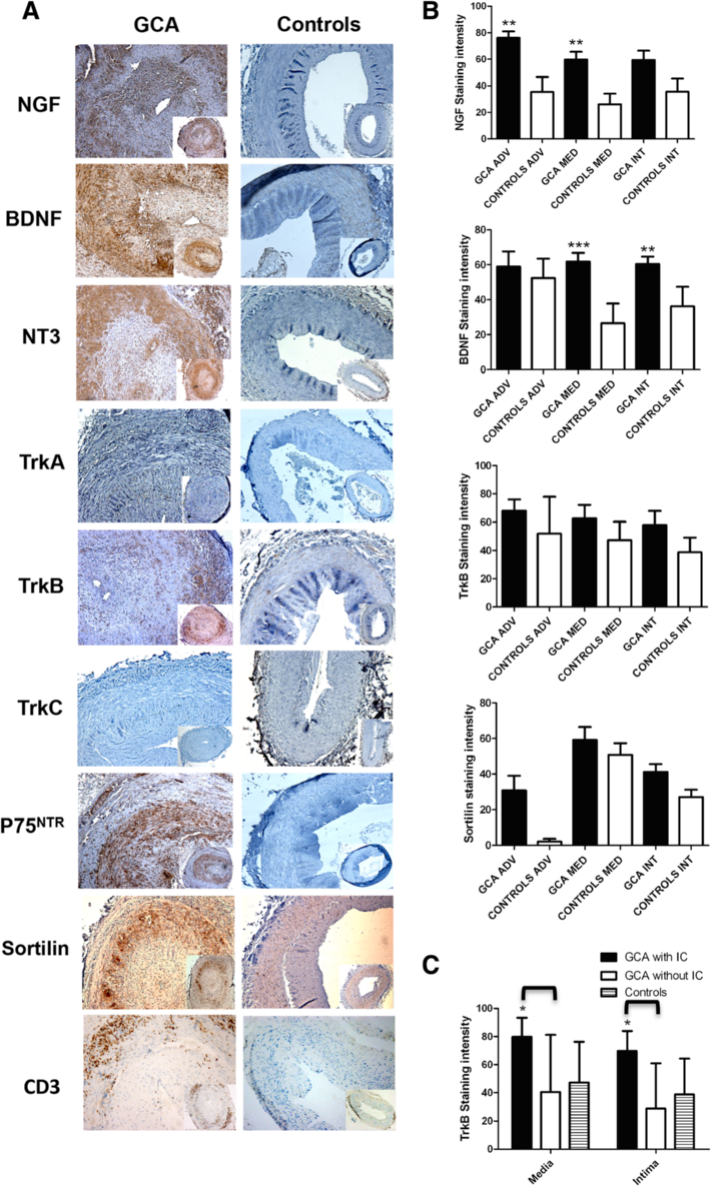
**Immunostaining of NTs and their receptors and T lymphocytes (CD3+) in temporal arteries of patients and controls. (A)** Panels are representative examples of immunohistological images (x50 and x200) of temporal arteries from GCA patients (*n* = 22) compared to controls (*n* = 21). **(B)**. Panels are measurements of significant NT immunostaining intensities. Results are shown as mean values and standard errors of the mean (SEM). **(C)** TrkB immunostaining in GCA patients with or without ischaemic complications. Immunoreactivity to TrkB in the media and intima of TAs in GCA patients with (*n* = 9) or without (*n* = 13) ischaemic complications. GCA, giant cell arteritis; NT, neurotrophin; TA, temporal artery; Trk, tropomyosin receptor kinase.

TAs from 22 GCA patients and 21 controls expressed NGF, BDNF, TrkB and sortilin, whereas TrkA and TrkC were not detected in any studied TAs (Figure 1A). However, significant differences in the intensity and location of staining were observed between GCA patients and controls. Indeed, NGF expression was more intense in the adventitia (76.2 +/- 19 vs. 35.4 +/- 22%, *P* = 0.002) and the media (59.6 +/- 25% vs. 26 +/- 22%, P = 0.004), respectively. BDNF was markedly expressed in the media (82 +/- 21 vs. 26.5 +/- 27%, *P* <0.0001) and the intima (78 +/- 20 vs. 36 ± 27%, *P* = 0.0007), respectively. Concerning sortilin, expression was prominent in the adventitia (31 ± 28 vs. 2 ± 4%, *P* = 0.003) and the intima (41 ± 14 vs. 27 ± 14%, *P* = 0.02), respectively (Figure 1B). Interestingly, only GCA arteries strongly expressed NT-3 and P75^NTR^ in all artery layers (Figure 1A).

By contrast, T lymphocytes (anti-CD3+) staining was localized in the media intima junction.

To search for a relationship between these expressions and the course of the disease, we evaluated the staining of NTs and NTRs in association with the clinical parameters of the 22 GCA patients (Table 1).

### TrkB is overexpressed in temporal arteries of GCA patients with ischaemic complications

Strikingly, only GCA patients with ischaemic events (stroke, amaurosis or permanent visual loss) (*n* = 9) had significant overexpression of TrkB in the media (*P* = 0.04) and intima (*P* = 0.015), compared to GCA patients without ischaemia (*n* = 13) (Figure 1C). No difference in expression of NTs and NTRs was found in the TAs of GCA patients regarding other clinical characteristics or inflammatory biomarkers. Moreover, we did not find any difference in immunostaining of NTs and NTRs between GCA patients who received corticosteroids before a TAB (*n* = 14) and untreated patients (*n* = 8).

Considering these differences in the expression of NTs and NTRs on *ex vivo* TAs in GCA patients, we achieved primary cultures for six TAs from biopsy-proven GCA patients and from 10 suspected GCA patients who had normal histological TAs as controls.

### Expression of neurotrophins and receptors in TASMCs from the temporal arteries of GCA patients and controls

**Figure 2:**
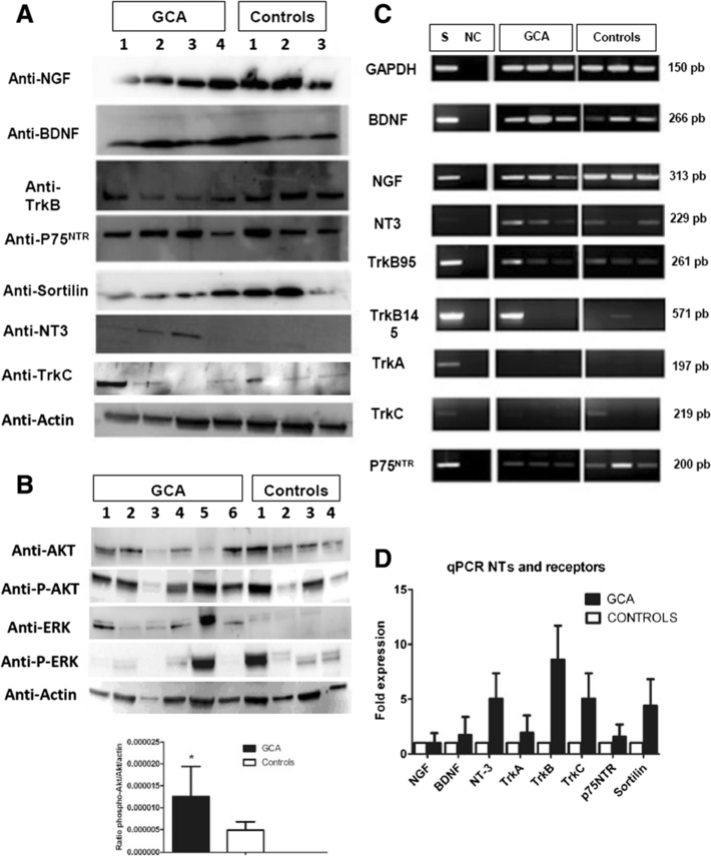
**Western blot (WB) analysis of GCA patients’ and controls’ TASMC lysates.** WB analysis of GCA patients’ (*n* = 6) and controls’ (*n* = 4) TASMC lysates for NTs and NT receptors **(A)** and for Akt, phospho-Akt, ERK 1/2 and phospho-ERK 1/2 and histograms for phospho-Akt /Akt/ actin ratio **(B)**. Panels are representative examples of WB analysis. **(C)** Transcription of NTs and NT receptors by TASMCs from GCA patients (*n* = 6) and controls (*n* = 6) cultured with 10% FCS. The neuroblastoma cell line SH-SY-5Y (S) was used as a positive control. Constitutively expressed GAPDH is a control of PCR efficiency. Panels are representative examples of the transcription assay. NC: negative control. **(D)** qRT-PCR analysis of NT and NT receptor expression in TASMC cultures from GCA patients (*n* = 6) and controls (*n* = 6), normalized to *HPRT* gene expression. Akt, protein kinase B; ERK, extracellular signal-regulated kinase FCS, foetal calf serum; GCA, giant-cell arteritis; NGF, nerve growth factor; NT, neurotrophin; PCR, polymerase chain reaction; TASMC, temporal artery VSMC; VSMC, vascular smooth muscle cell.

Protein expression was assessed by western blotting in proteins extracted from the TASMCs of GCA patients and controls (Figure 2A). Whereas NGF, BDNF, TrkB, TrkC, p75^NTR^ and sortilin were detected in TASMCs from patients and controls, proteins for TrkA receptor were not detected in cells from either of these groups. In contrast, NT-3 protein was only detected in GCA and not in control cells. Exploration of signalling pathways at baseline showed that phospho-Akt is predominantly detected in GCA-derived TASMCs than control. The ratio of phospho-Akt/ Akt/actin is significantly higher in GCA cells patients in comparison to patients controls (*P* = 0.033) (Figure 2B) whereas no difference was found for ERK 1/2 and phospho-ERK 1/2. In parallel, we have confirmed by RT-PCR that the corresponding mRNAs for NGF, BDNF, TrkB (truncated 95 length) and p75^NTR^ were present but not those for TrkA, as expected (Figure 2B). However, some discrepancies were noted between protein and mRNA expressions. This was evidenced for NT-3 mRNA, which was detected in all sampled cells from patients and controls, and feebly for the TrkC transcript. Therefore, a quantitative RT-PCR was performed to determine the differences between transcript expression of NT and NTR genes in cells from patients and controls.

While BDNF, NGF and NT-3 mRNA were expressed, those of NT-3, TrkB, TrkC and sortilin were overexpressed (increase by fivefold) in TASMCs from GCA patients compared to controls, but the difference was not significant (Figure 2C). Thus, because NGF and BDNF and their receptors were detected in TASMCs from GCA and control patients, the functional effects of these two NTs were evaluated in cultured cells from TAs from the two groups and compared with PDGF as a positive control.

### Proliferation effects of NT and NT receptor blockers in GCA patients

**Figure 3:**
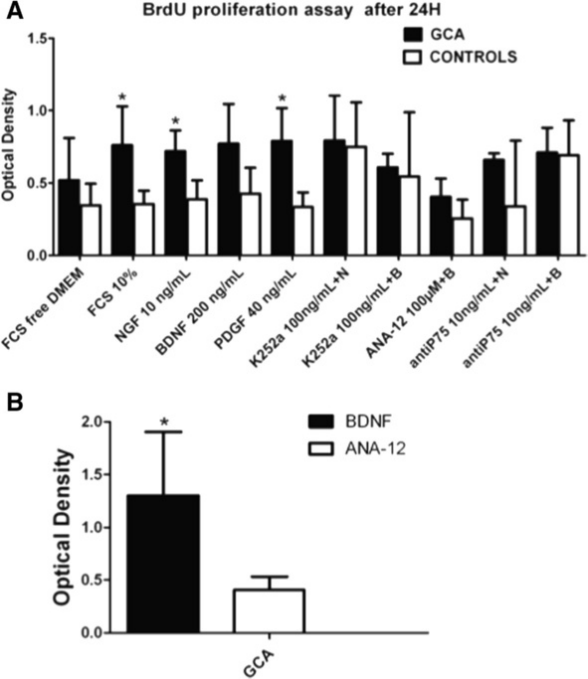
**Effects of NTs and the inhibitors of Trk (K252a), TrkB (ANA-12) and p75** ^**NTR**^ **(anti-p75) on TASMC proliferation in GCA patients and controls.** Proliferation assay performed with a BrdU assay on day 1 **(A)** in serum-starved TASMC incubated with NT or NT receptor inhibitors, or a combination of NT and NT receptor inhibitors, in GCA patients (*n* = 6) and controls (*n* = 10). In all figures, bars represent the mean of three experiments with their SEMs. K252a: specific Trk receptor inhibitor; ANA-12: a specific TrkB receptor inhibitor; anti-p75: p75^NTR^ Ab inhibitor; N (NGF) or B (BDNF) + K (K252a) or anti-p75^NTR^: NGF or BDNF are added at the same time with the specific inhibitor. **(B)** Effect of ANA-12 on GCA-derived TASMCs proliferation. Three independent assays were performed and cells were seeded in triplicate for each cell line. BDNF, brain-derived neurotrophic factor; GCA, giant-cell arteritis; NGF, nerve growth factor; NT, neurotrophin; SEM, standard errors of the mean; TASMC, temporal artery VSMC; Trk, tropomyosin receptor kinase; VSMC, vascular smooth muscle cell.

NGF promoted proliferation of TASMCs from GCA patients compared to controls (*P* = 0.025) similarly to FCS (*P* = 0.012) and PGDF (*P* = 0.023) by day 1. BDNF tended to enhance cell proliferation by day 1 especially in TASMCs from GCA patients (*P* = 0.052) (Figure 3A). Proliferation effects with NTs, FCS 10% and PDGF tended to disappear by day 3 and day 4 with no difference between the two groups (Additional file 4). Addition of K252a, ANA-12 or anti-p75^NTR^ Ab prior NTs did not induce a significant difference between the two groups. However, ANA-12 significantly decreased proliferation of TASMCs from GCA in the presence of BDNF (*P* = 0.010), demonstrating that BDNF-induced proliferation was depending on TrkB activation (Figure 3B).

Given that NGF and, to a lesser extent, BDNF induced proliferation in TASMCs from GCA patients compared to controls, we searched for the potential effect of NTs in TASMC invasion properties in Matrigel chambers to determine whether NTs are involved in intimal hyperplasia as shown by immunohistochemistry.

### BDNF activates TASMC migration in GCA patients but not in controls

**Figure 4:**
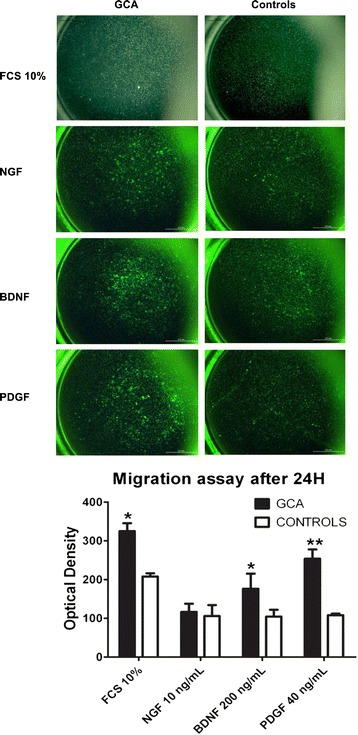
**Migration assay of TASMC with NGF and BDNF.** Migration assays were performed with TASMCs from GCA patients (*n* = 6) and controls (*n* = 6) incubated in the upper chamber with serum-free DMEM during 48 h whereas NGF (10 ng/mL), BDNF (200 ng/mL), PDGF (40 ng/mL) or 10% FCS were added in the lower chamber during 24 h. Top: representative images of the lower chamber after migration and fluorescent staining. Bottom: data analysis obtained from two independent assays performed with cells seeded in duplicate for each cell line. BDNF, brain-derived neurotrophic factor; DMEM, Dulbecco’s modified Eagle’s medium; FCS, foetal calf serum; GCA, giant cell arteritis; NGF, nerve growth factor; PDGF, platelet-derived growth factor; TASMC, temporal artery VSMC; VSMC, vascular smooth muscle cell.

Comparison of TASMC invasion in GCA and control patients demonstrated that TASMCs from GCA patients were significantly able to migrate in the presence of exogenous BDNF compared to those from controls (*P* = 0.012) after a 24-h exposure (Figure 4). In contrast, no effect was shown for exogenous NGF whatever the status of TASMCs in patients or controls. Of note, only cells from GCA patients were able to significantly invade Matrigel chambers in the presence of 10% FCS (*P* = 0.0175, compared to cells from controls) as was also observed for exogenous PDGF (*P* <0.0001 vs. cell migration in controls). Strikingly, neither BDNF nor PDGF induced migration of TASMCs in controls. Migration of TASMCs in GCA patients was only detected after a 24-h exposure and disappeared after 48 h of incubation (data not shown).

Because NTs are expressed in TAs and in VSMCs of GCA patients, we looked for secretions by TASMC cells in cultures and the sera.

### NGF concentration was higher in the sera and in TASMC culture-supernatants from GCA patients

**Figure 5:**
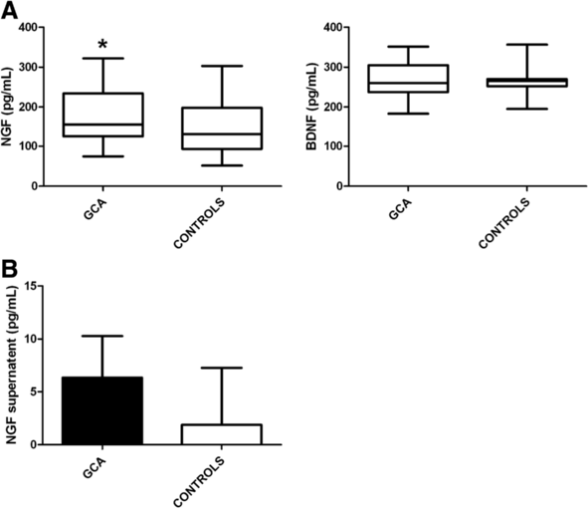
**Sera and supernatant concentrations of NGF and BDNF from GCA patients and controls. (A)** NGF and BDNF sera concentrations from patients with GCA (*n* = 30) compared to controls (*n* = 48) with a sera dilution of 1:100. **(B)** NGF TASMC-related supernatant concentration from GCA patients (*n* = 8) compared to controls (*n* = 8). Supernatants were collected when VSMC reached 80% confluence, between passages 3 and 5. Experiments were performed in duplicate, with three independent ELISA assays and data are presented as pg/mL. BDNF, brain-derived neurotrophic factor; ELISA, enzyme-linked immunosorbent assay; GCA, giant cell arteritis; NGF, nerve growth factor; TASMC, temporal artery VSMC; VSMC, vascular smooth muscle cell.

NGF sera concentrations were significantly higher in GCA patients compared to controls (177 +/- 67.6 vs. 145.5 +/- 66 pg/mL, *P* = 0.04) (Figure 5A) whereas no difference was found for BDNF (Figure 5A) or NT-3 (data not shown). There was no correlation between NGF*,* BDNF or NT-3 level and ischaemic events or inflammatory syndrome. NGF and BDNF secretion was measured in cell supernatants. NGF levels tended to be higher in TASMCs from GCA patients (*P* = 0.06) (Figure 5B) whereas no difference was detected for BDNF and NT-3 supernatants (data not shown).

## Discussion

Despite substantial improvement in understanding the pathophysiologic features of GCA, some mechanisms still remain unclear, especially in relation to vascular remodelling. Herein, we report on the expression and functions of NTs and their receptors, which are potentially involved in vascular remodelling in GCA. It is worth noting that NGF, BDNF and sortilin are significantly overexpressed in different histological layers of TAs in GCA patients compared to those of controls. In addition, TrkB staining was significantly higher in the TAs of GCA patients with a cranial ischaemic event. TrkA and TrkC were not detected in patients or controls, whereas NT-3 and p75^NTR^ were enhanced only in GCA patients. These data dealt with mRNA and protein expression of NTs and NTRs in TASMCs from GCA patients and controls. Enhanced TrkB transcription was observed in GCA cells whereas those of TrkA and TrkC were feebly detected.

The expressions of NGF, BDNF and sortilin in the TAs of GCA patients could be related to other inflammatory disorders associated with localized expression of NTs. Indeed, NGF is expressed in endothelial cells and keratinocytes in patients with psoriasis [12], and NGF and BDNF are present in the inflamed joints of patients with rheumatoid arthritis [13,14]. Of note, constitutive NGF expression and release in both human and mouse articular chondrocytes is enhanced by interleukin (IL)-1β [15]. Likewise, NTs are detected in other bronchial smooth muscle cells during asthma [16] or sarcoidosis [17] and their relationship with inflammatory cytokines, IL-1β and interferon (IFN)-γ has been established by the induction of NGF, and BDNF mRNA expression and secretion in cell-culture supernatants [18,19]. Hence, the overexpression of NTs and their receptors in TAs from GCA patients could be, in part, related to the presence of proinflammatory cytokines in all layers of the inflamed arterial wall. Moreover, it has been demonstrated that ischaemia contributes to the overexpression of some NTs and their related receptors, as we have shown with the increased TrkB expression in GCA patients with a cranial ischaemic event. Indeed, in experimental arterial injury that leads to ischaemia, enhanced expression of NGF, BDNF and their specific receptors has been described [3]. Furthermore, in a murine experimental model of atherosclerosis, the TrkB receptor is overexpressed in neointimal VSMC cells whereas decreased TrkB expression reduces atherosclerotic lesions [20].

Moreover, sortilin expression is enhanced in the adventitia and intima of TAs from GCA patients. This data could be correlated with intimal TrkB overexpression, as sortilin acts as an intracellular protein transporter for immature NTs [21] and controls the regulation of BDNF trafficking and release [22]. Such a function could be related to BDNF and sortilin overexpression in TAs from GCA patients. In addition, sortilin is also a co-receptor for p75^NTR^, which was only observed in the TAs of GCA patients. p75^NTR^ is mostly absent in healthy controls (HCs): its expression is enhanced by pathologic conditions such as diabetes or atherosclerosis [3,5,23].

Serum NGF levels are significantly enhanced in GCA patients, but not BDNF or NT-3. Such elevated NGF levels have been previously reported in inflammatory diseases such as Sjögren syndrome [24].

The proliferation effects of NTs are well described in smooth muscle cells other than VSMCs, especially for NGF and BDNF [25-28]. Given that isolation and expansion in the culture of smooth muscle cells from temporal arteries was previously reported [29], functional studies of TASMCs isolated from GCA and controls were performed in our study. Indeed, NGF promoted proliferation in GCA-derived TASMCs similarly to PDGF. Because TrkA was not detected in TASMCs, this effect probably took place through p75^NTR^ activation as already described in brain tumor-initiating cells [30]. BDNF induced GCA-derived TASMCs proliferation depending on TrkB as demonstrated by ANA-12, a specific inhibitor of TrkB [10]. This effect, well reported in smooth muscle cells in pulmonary hypertension [27] is not significant probably because of our small number samples. In GCA patients we have demonstrated that BDNF promotes TASMCs migration rather than their proliferation. Because both TrkB and BDNF were prominently expressed in the same areas of GCA temporal arteries, it is probable that BDNF activates TASMCs migration from TAs of patients through its interaction with TrkB. Our data are consistent with previous studies on the effects of VSMCs on NTs [3,4,31]. Indeed, BDNF-related migration was comparable to that observed with PDGF, as described previously, [4] and was more consistent than that observed with NGF.

Exploration of signalling pathways in our study showed that PI3/Akt pathway seemed to be more activated than ERK1/2 pathway in GCA patients compared to controls. Though PI3/Akt signalling pathway can be activated by TNF-α in human smooth muscle cells [32] or IL-1β in rat brain astrocytes [33], our results are consistent with BDNF-induced migration in human chondrosarcoma [34] through a transducing signal involving TrkB.

## Conclusions

Neurotrophins inducing proliferation and migration of VSMC are potentially implicated in vascular remodelling in GCA.

**Figure 6:**
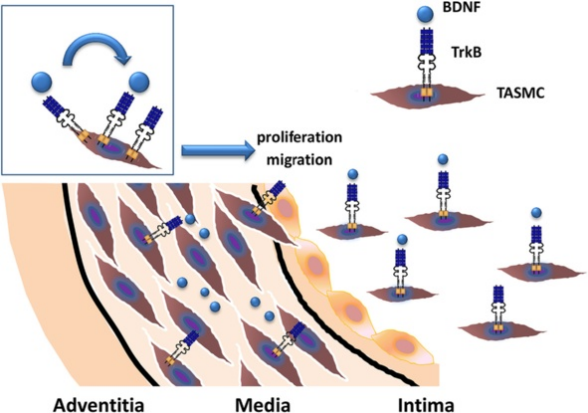
**Fine-tuning role of BDNF and TrkB in proliferation and migration of TASMC.** BDNF, brain-derived neurotrophic factor; TrkB, tropomyosin receptor kinase B; TASMC, temporal artery vascular smooth muscle cell.

Taken together, our results provide new insights regarding the involvement of NTs and NTRs in the vascular remodelling of GCA pathogenesis. Their overexpression in TAs from GCA patients could facilitate VSMC proliferation and migration from media to intima, thus contributing to intimal hyperplasia. Particularly, a fine-tuning role of the BDNF/TrkB axis is implicated in proliferation and migration of VSMC from GCA patients (and not from controls) as demonstrated *in vitro* (Figure 6). However, further studies are needed to elucidate the signalling pathways that allow VSMC migration in order to define new therapeutic approaches for patients with GCA.

## Additional files

Additional file 1:
**VSMC phenotype in immunocytochemistry assay.**
Additional file 2:
**Primers used in qPCR and RT-PCR studies.**
Additional file 3:
**Dose-response of NT and NT receptor inhibitors.**
Additional file 4:
**Proliferation assays on days 3 and 4.**

## Abbreviations

Ab: antibody
ACR: American College of Rheumatology
Akt: protein kinase B
BDNF: brain-derived neurotrophic factor
DMEM: Dulbecco’s modified Eagle’s medium
ELISA: enzyme-linked immunosorbent assay
ERK: extracellular signal-regulated kinase
FCS: foetal calf serum
FGF: fibroblast growth factor
GCA: giant cell arteritis
HC: healthy control
IL: interleukin
INF: interferon
NGF: nerve growth factor
NT: neurotrophin
NT-3: neurotrophin-3
NTR: neurotrophin receptor
PCR: polymerase chain reaction
PDGF: platelet-derived growth factor
RT: reverse transcriptase
SEM: standard errors of the mean
TA: temporal artery
TAB: temporal artery biopsy
TASMC: temporal artery VSMC
TNF-α: tumour necrosis factor alpha
Trk: tropomyosin receptor kinase
VSMC: vascular smooth muscle cell
