# Supplementary figures and images for: Neurotrophins are expressed in giant cell arteritis lesions and may contribute to vascular remodeling
Source: Arthritis Res Ther. 2014 Nov 24;16(6):487. doi: 10.1186/s13075-014-0487-z (PMC4274683; doi:10.1186/s13075-014-0487-z)

**Additional file 1. VSMC phenotype in immunocytochemistry assay**

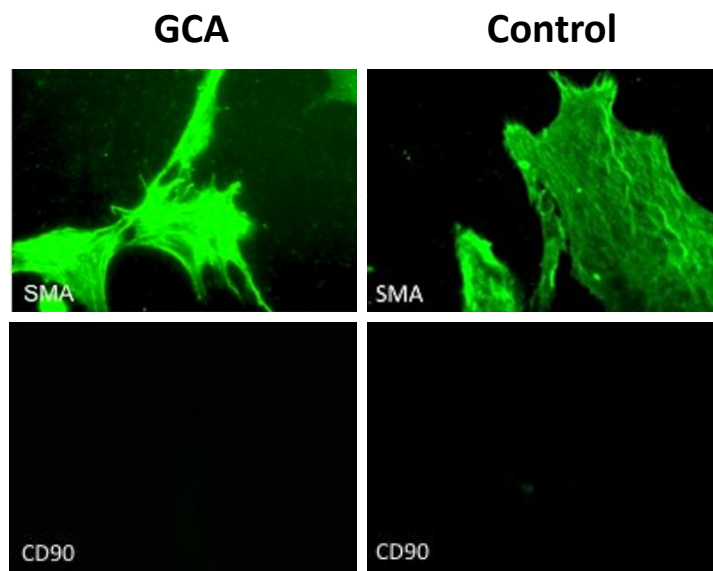

Supplement: Additional file 1: — VSMC phenotype in immunocytochemistry assay. [file 13075_2014_487_MOESM1_ESM.pdf]

Additional file 3. Dose-response of NT and NT receptor inhibitors

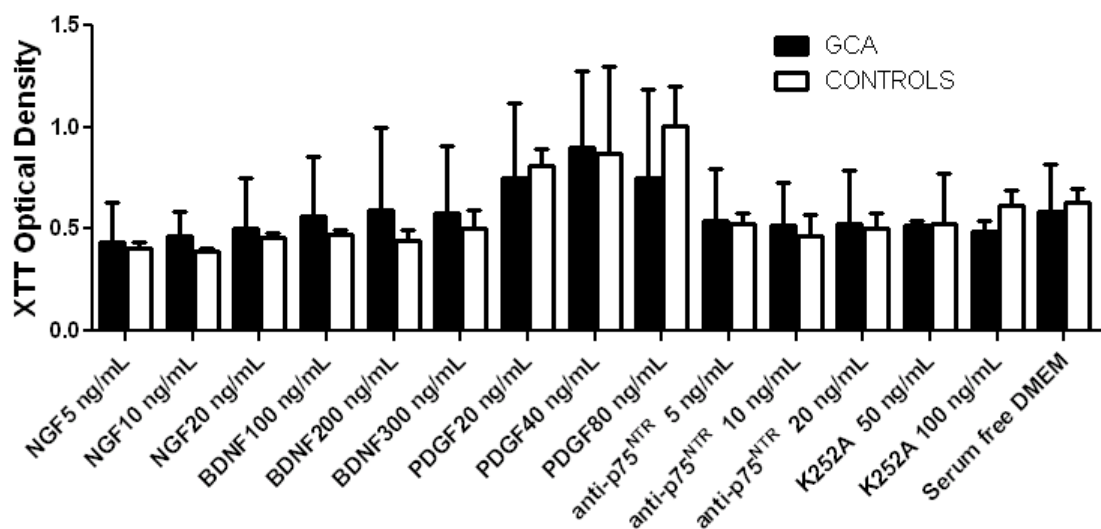

Supplement: Additional file 3: — Dose-response of NT and NT receptor inhibitors. [file 13075_2014_487_MOESM3_ESM.pdf]

Additional file 4: Proliferation assays on days 3 and 4

A

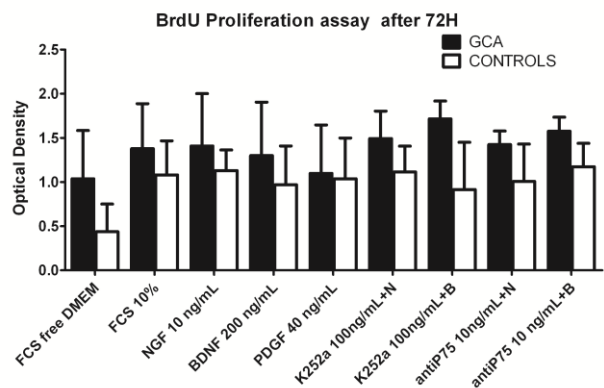

B

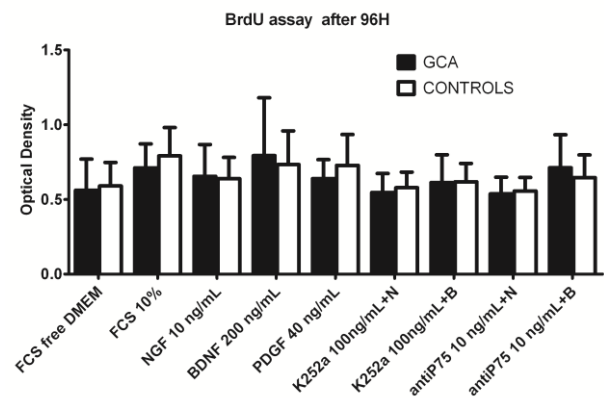

Supplement: Additional file 4: — Proliferation assays on days 3 and 4. [file 13075_2014_487_MOESM4_ESM.pdf]
