# Supplementary material for: Neurotrophins are expressed in giant cell arteritis lesions and may contribute to vascular remodeling
Source: Arthritis Res Ther. 2014 Nov 24;16(6):487. doi: 10.1186/s13075-014-0487-z (PMC4274683; doi:10.1186/s13075-014-0487-z)
Supplement: Additional file 2: — Primers used in qPCR and RT-PCR studies. [file 13075_2014_487_MOESM2_ESM.pdf]

Additional file 2. Primers used in qPCR and RT-PCR studies

Genes	Accession number	qPCR Primers (3′6FAM-MGBNFQ5’)	RT-PCR primers	Length (bp)	TM (°C)
NGF	NM_002506.2	Hs01113193_m1	**F**: ATACAGGCGGAACCACACTC<br>**R**: TGCTCCTGTGAGTCCTGTTG	**313**	**58**
BDNF	NM_001143810	Hs.00380947_m1	**F**:TACTTTGGTTGCATGAAGGCTCC<br>**R**:ACTTGACTACTGAGCATCACCCTG	**266**	**58**
NT-3	NM_001102654	Hs00267375_s1	**F**: TGGCATCCAAGGTAACAACA<br>**R**: GGTGTCCATTGCAATCACTG	**229**	**58**
TrkA	NM_002507	Hs01021011_m1	**F:** TCAACAAATGTGGACGGAGA<br>**R:** GTGGTGAACACAGGCATCAC	**197**	**58**
TrkB	NM_001018064.1	Hs.01093096_m1	**TrkB145**<br>**F:** AGGGCAACCCGCCCACGGAA<br>**R:** GGATCGGTCTGGGGAAAAG	**571**	**62**
			**TrkB 95**<br>**F:**GTTTCATAAGATCCCACTGGA<br>**R:**TGCTGCTTAGCTGCCTGAGAG	**261**	**58**
TrkC	NM_001243101	Hs00176797_m1	**F:** ACTTCCGTCAGGGACACAAC<br>**R:** CCTCCCTCTGGAAATCCTTC	**219**	**58**
$p75^{NTR}$	NM_002507	Hs.00609976_m1	**F:** GTGGGACAGAGTCTGGGTGT<br>**R:** AAGGAGGGGAGGTGATAGGA	**200**	**58**
Sortilin	NM_002959	Hs.00907094_m1	**F:** GCTGGTCACAGTCGTAGCAG<br>**R:** TTAGTGTGGGAGGCTGTGTC	**150**	**60**
GAPDH			**F:**GGGTGGAATCATATTGGAACATG<br>**R:** GTCGGAGTCAACGGATTTGG	**150**	**58**
HPRT	NM_000194	Hs.02800695_m1			

F: Forward, R: Reverse
